# Proper Distance Metrics for Phylogenetic Analysis Using Complete Genomes without Sequence Alignment

**DOI:** 10.3390/ijms11031141

**Published:** 2010-03-18

**Authors:** Zu-Guo Yu, Xiao-Wen Zhan, Guo-Sheng Han, Roger W. Wang, Vo Anh, Ka Hou Chu

**Affiliations:** 1 School of Mathematics and Computational Science, Xiangtan University, Hunan 411105, China; E-Mails: zhan031001140604@163.com (X.-W.Z.); korea10282003@163.com (G.-S.H.); 2 School of Mathematical Sciences, Queensland University of Technology, GPO Box 2434, Brisbane, QLD 4001, Australia; E-Mail: v.anh@qut.edu.au (V.A.); 3 Department of Mathematics, Chinese University of Hong Kong, Shatin, N.T., Hong Kong, China; E-Mail: wwang_00@yahoo.com (R.W.W.); 4 Department of Biology, Chinese University of Hong Kong, Shatin, N.T., Hong Kong, China; E-Mail: kahouchu@cuhk.edu.hk (K.H.C.)

**Keywords:** phylogenetic analysis, complete genome, composition vector, correlation-related distance metric

## Abstract

A shortcoming of most correlation distance methods based on the composition vectors without alignment developed for phylogenetic analysis using complete genomes is that the “distances” are not proper distance metrics in the strict mathematical sense. In this paper we propose two new correlation-related distance metrics to replace the old one in our dynamical language approach. Four genome datasets are employed to evaluate the effects of this replacement from a biological point of view. We find that the two proper distance metrics yield trees with the same or similar topologies as/to those using the old “distance” and agree with the tree of life based on 16S rRNA in a majority of the basic branches. Hence the two proper correlation-related distance metrics proposed here improve our dynamical language approach for phylogenetic analysis.

## 1. Introduction

Whole genome sequences are generally accepted as excellent tools for studying evolutionary relationships [1]. Traditional distance methods with multiple alignment or various sequence evolutionary models for phylogenetic analysis are not directly applicable to the analysis of complete genomes.

A number of methods without sequence alignment for deriving species phylogeny based on overall similarities of complete genomes have been developed. These include fractal analysis [2–4], dynamical language model [5], information-based analysis [6–8], log-correlation distance and Fourier transformation with Kullback-Leibler divergence distance [9], Markov model [10–15], principal component analysis [16] and singular value decomposition (SVD) [17–19]. The analyses based on the Markov model and dynamical language model without sequence alignment using 103 prokaryotes and 6 eukaryotes have yielded trees separating the three domains of life, Archaea, Eubacteria and Eukarya, with the relationships among the taxa consistent with those based on traditional analyses [5,11]. These two methods were also used to analyze the complete chloroplast genomes [5,12]. The SVD method was used to analyze mitochondrial genomes of 64 selected vertebrates [19]. A correlation-distance method without removing the random background (similar to [7]) was used to analyze rRNA gene sequences as DNA barcodes [20].

In the above approaches of SVD, Markov model and dynamical language model, there is a step to calculate the correlation-related distance between two genomes after removing the randomness or noise from the composition vectors. A drawback is that these correlation-related distances are not proper distance metrics in the strict mathematical sense (Professor Bailin Hao, personal communication, 2009; see also [21]). There are some ways to overcome this problem. One way is to change the concept of distance to that of dissimilarity proposed by Xu and Hao [15] in the Markov model approach. Another way is to replace a pseudo-distance by a proper distance metric, which requires that the results are not worsened from the biological point of view. In the first way, there is no widely accepted mathematical definition for the concept of dissimilarity or similarity. Chen *et al.* [22] defined a similarity metric, but unfortunately the sample correlation between two vectors in a vector space does not yield a proper similarity under their definition.

In this paper, we follow the second way and propose two proper correlation-related distance metrics to replace the pseudo-distance in the dynamical language approach used by Yu *et al*. [5]. We then evaluate the effects of this replacement on the analysis of a wide range of complete genomes from the biological point of view.

## 2. Dynamical Language Approach for Phylogenetic Analysis

Three kinds of data from the complete genomes can be analysed using the dynamical language approach proposed by Yu *et al*. [5]. They are the whole DNA sequences (including protein-coding and non-coding regions), all protein-coding DNA sequences and the amino acid sequences of all protein-coding genes. We outline this approach here.

There are a total of *N* = 4*^K^* (for DNA sequences) or 20*^K^* (for protein sequences) possible types of *K*-strings, that is, the strings with fixed length *K*. We denote the length of a DNA or protein sequence as *L*. Then a window of length *K* is used to slide through the sequences by shifting one position at a time to determine the frequencies of each of the *N* kinds of *K*-strings in this sequence. We define *p*(*α*_1_*α*_2_...*α_K_*) = *n*(*α*_1_*α*_2_...*α_K_*) / (*L* – *K* + 1) as the observed frequency of a *K* -string *α*_1_*α*_2_...*α_K_*, where *n*(*α*_1_*α*_2_...*α_K_*) is the number of times that *α*_1_*α*_2_...*α_K_* appears in this sequence. For the DNA or amino acid sequences of the protein-coding genes, denoting by *m* the number of protein-coding genes from each complete genome, we define $\left(\sum_{j=1}^{m}n_{j}(\alpha_{1}\alpha_{2}\ldots \alpha_{K}))/(\sum_{j=1}^{m}\left(L_{j}-K+1\right)\right)$ as the observed frequency of a *K*-string *α*_1_*α*_2_...*α_K_*; here *n_j_* (*α*_1_*α*_2_...*α_K_*) means the number of times that *α*_1_*α*_2_...*α_K_* appears in the *j*th protein-coding DNA sequence or protein sequence, and *L_j_* the length of the *j*th sequence in this complete genome. Then we can form a *composition vector* for a genome using *p*(*α*_1_*α*_2_...*α_K_*) as components for all possible *K*-strings *α*_1_*α*_2_...*α_K_*. We use *p_i_* to denote the *i*-th component corresponding to the string type *i*, *i* = 1,…,*N* (*N* strings are arranged in a fixed order as the alphabetical order). In this way we construct a composition vector *p* = (*p*_1_, *p*_2_,..., *p_N_*) for a genome.

Yu *et al*. [5] considered an idea from the theory of dynamical language [23] that a *K*-string *s*_1_*s*_2_...*s_K_* is possibly constructed by adding a letter *s_K_* to the end of the (*K* – 1) -string *s*_1_*s*_2_...*s*_*K*–1_ or a letter *s*_1_ to the beginning of the (*K* – 1) -string *s*_2_*s*_3_...*s_K_*. After counting the observed frequencies for all strings of length (*K* – 1) and the four or 20 kinds of letters, the expected frequency of appearance of *K*-strings is predicted by:

(1) $$q(s_{1}s_{2}\ldots s_{K})=\frac{p(s_{1}s_{2}\ldots s_{K-1})p(s_{K})+p(s_{1})p(s_{2}s_{3}\ldots s_{K})}{2}$$

where *p*(*s*_1_) and *p*(*s_K_*) are frequencies of nucleotides or amino acids *s*_1_ and *s_K_* appearing in this genome. Then *q*(*s*_1_*s*_2_...*s_K_*) of all 4*^K^* or 20*^K^* kinds of *K*-strings is viewed as the noise background. We then subtract the noise background before performing a cross-correlation analysis through defining:

(2) $$X(s_{1}s_{2}\ldots s_{K})=\{\begin{array}{ccc} p(s_{1}s_{2}\ldots s_{K})/q(s_{1}s_{2}\ldots s_{K})-1, & if & q(s_{1}s_{2}\ldots s_{K})\neq 0,\\ 0, & if & q(s_{1}s_{2}\ldots s_{K})=0, \end{array}$$

The transformation *X* = (*p* / *q*) – 1 has the desired effect of subtraction of random background in *p* and rendering it a stationary time series suitable for subsequent cross-correlation analysis.

Then we use *X* (*s*_1_*s*_2_...*s_K_*) for all possible *K*-strings *s*_1_*s*_2_...*s_K_* as components and arrange according to a fixed alphabetical order all the *K*-strings to form a composition vector *X* = (*X*_1_, *X*_2_,..., *X_N_*) for genome *X*, and likewise *Y* = (*Y*_1_, *Y*_2_,...,*Y_N_*) for genome *Y*.

Then we view the *N* components in the vectors *X* and *Y* as samples of two random variables respectively. The sample correlation *C*(*X*, *Y*) between any two genomes *X* and *Y* is defined in the usual way in probability theory as:

$$C(X,Y)=\frac{\sum_{i=1}^{N}X_{i}Y_{i}}{{\left(\sum_{i=1}^{N}X_{i}^{2}\sum_{i=1}^{N}Y_{i}^{2}\right)}^{\frac{1}{2}}}$$

The distance *D_r_* (*X*, *Y*) between the two genomes is then defined by *D_r_* (*X*, *Y*) = (1 – *C*(*X*, *Y*)) / 2. A distance matrix for all the genomes under study is then generated for the construction of phylogenetic trees. This distance method to construct phylogenetic tree is referred to as the *dynamical language model method* [5]. Finally, we construct all trees using the neighbour-joining (NJ) method [24] in the software *SplitsTree4* V4.10 [25] or in the *Molecular Evolutionary Genetics Analysis* software (MEGA 4) [26] based on the distance matrices.

**Figure 1.**
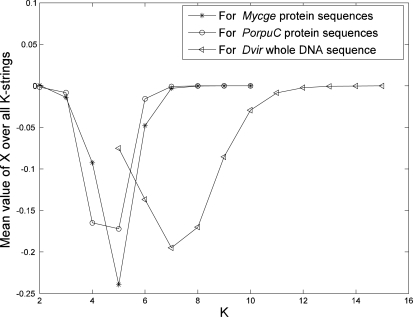
The plot of mean value of *X* over all *K*-strings as a function of *K*. The abbreviations “Mycge”, “PorpuC” and Dvir” are one of genomes in our first three datasets.

To determine a best length of strings (*K*) in our model, we plot the mean value of X over all *K*-strings from a genome (whole DNA sequences or protein sequences) as a function of *K* (see Figure 1 for examples from our data). The mean value of *X* starts to approach zero at *K* = 6 or 7 if we use protein sequences from genome and at *K* = 11 or 12 if we use whole DNA sequence. The mean value of *X* being close to zero means that the value of *p* (from the sequence) is almost equal to value of *q* (from the model). Hence these *K* values are suitable for phylogeny reconstruction using our approach. This result is also confirmed later in this paper from a biological point of view.

## 3. Proper Distance Metrics in Vector Spaces

Each genome can be considered as a point in *N* = 4*^K^* (for DNA sequences) or 20*^K^* (for protein sequences) dimensional space represented by its composition vector *X* = (*X*_1_, *X*_2_,..., *X_N_*).

A function *D*(*X*, *Y*) between two vectors *X* and *Y* is said to be a distance metric if it satisfies the following properties:

- *D*(*X*, *Y*) ≥ 0; and *D*(*X*, *Y*) = 0 if and only if *X* = *Y*;
- *D*(*X*, *Y*) = *D*(*Y*, *X*);
- *D*(*X*, *Z*) ≤ *D*(*X*, *Y*) + *D*(*Y*, *Z*) for any *X*, *Y* and *Z*.

The inequality (iii) is called the *triangle inequality*. A distance metric *D*(*X*, *Y*) is said to be normalized if 0 ≤ *D*(*X*, *Y*) ≤ 1 for any *X* and *Y*.

If we denote:

$$X_{u}=\frac{X}{|X|,}\;Y_{u}=\frac{Y}{\left|Y\right|}$$

where |*X*| and |*Y*| are the lengths of the vectors *X* and *Y* respectively, then *X_u_* and *Y_u_* are unit vectors (*i.e.*, have length 1). Let *θ* be the angle between two vectors of *X* and *Y*. It is well known that *C*(*X_u_*, *Y_u_*) = cos*θ*.

The distance defined by *D_r_* (*X*, *Y*) = (1 – *C*(*X*, *Y*)) / 2 is not a proper distance metric because it does not satisfy condition (i) (except for unit vectors) and the triangle inequality (iii) [21]. In the following we describe two proper distance metrics related to the sample correlation.

### 3.1. Chord Distance

The chord distance is defined on the set of unit vectors in a vector space as the length of the chord constructed from two unit vectors. Mathematically, let *X_u_* = (*X_u1_*, *X_u2_*,…,*X_uN_*) and *Y_u_* = (*Y_u1_*, *Y_u2_*,…, *Y_uN_*) be two unit vectors; then the chord distance *D_chord_* (*X_u_*, *Y_u_*)is defined as:

(3) $$\begin{array}{ll} D_{\text{chord}}(X_{u},\;Y_{u}) & =\sqrt{\sum_{i=1}^{N}{\left(X_{ui}-Y_{ui}\right)}^{2}}=\sqrt{\sum_{i=1}^{N}X_{ui}^{2}+\sum_{i=1}^{N}Y_{ui}^{2}-2\sum_{i=1}^{N}X_{ui}Y_{ui}}\\ & \;\;\;\;\;\;\;\;\;\;\;\;\;\;\;\;\;\;\;\;\;\;\;\;\;\;\;\;=\sqrt{2[1-C(X_{u},\;Y_{u})]}=\sqrt{2[1-C(X,\;Y)]} \end{array}$$

It is seen that *D_chord_* (*X_u_*, *Y_u_*) = 0 if and only if *C*(*X_u_*, *Y_u_*) = 1, *i.e.*, cos*θ*(*X_u_*, *Y_u_*) = 1, which implies that *θ*(*X_u_*, *Y_u_*) = 0 because the angle *θ*(*X_u_*, *Y_u_*) between the two vectors *X_u_* and *Y_u_* is in [0, *π*]. This result means that the two vectors *X_u_* and *Y_u_* are identical. It is obvious that *D_chord_* (*X_u_*, *Y_u_*) = *D_chord_* (*Y_u_*, *X_u_*). Because the three chords constructed by the pairs *X_u_* and *Y_u_*, *X_u_* and *Z_u_*, *Y_u_* and *Z_u_* are the three edges of a triangle, and the sum of the lengths of any two edges of a triangle is larger or equal to the length of the third edge, the triangle inequality of the chord distance follows. Hence the chord distance is a proper distance metric in the strict mathematical sense. The chord distance *D_chord_* (*X_u_*, *Y_u_*) can be normalized by $D_{\text{chord}}^{\text{norm}}(X_{u},\;Y_{u})=D_{\text{chord}}(X_{u},\;Y_{u})/2$. This distance is also called Cavalli-Sforza chord distance [27] or described on pp. 163–166 of [28]. This distance performed well in simulations of tree-building algorithms by Takezaki and Nei [29]. It has also been used to analyze microarray gene expression data [30].

### 3.2. Piecewise Distance

This distance metric is also defined on the set of unit vectors in a vector space. For any two unit vectors *X_u_* and *Y_u_*, we define:

(4) $$D_{\text{piecewise}}(X_{u},\;Y_{u})=\{\begin{array}{ccc} 1-C(X_{u},\;Y_{u})/\rho & if & C(X_{u},\;Y_{u})\neq 1\\ 0 & if & C(X_{u},\;Y_{u})=1 \end{array}$$

where *ρ* is any positive real number which is not smaller than 3. We call *D_piecewise_* (*X_u_*, *Y_u_*) the *piecewise distance*.

By definition, *D_piecewise_* (*X_u_*, *Y_u_*) = 0 if and only if *C*(*X_u_*, *Y_u_*) = 1, which means that the two vectors *X_u_* and *Y_u_* are identical as shown above. It is also obvious that *D_piecewise_* (*X_u_*, *Y_u_*) = *D_piecewise_* (*Y_u_*, *X_u_*). Using the facts *ρ* ≥ 3, −1 ≤ *C*(*X_u_*, *Y_u_*) ≤ 1 for any two unit vectors and *D_piecewise_* (*X_u_*, *Y_u_*) + *D_piecewise_* (*Y_u_*, *Z_u_*) – *D_piecewise_* (*X_u_*, *Z_u_*) = [*ρ* + *C*(*X_u_*, *Y_u_*) + *C*(*Y_u_*, *Z_u_*) –*C*(*X_u_*, *Z_u_*)]/*ρ* ≥ 0, we get the triangle inequality for the piecewise distance. Hence the piecewise distance is a proper distance metric in the strict mathematical sense. The piecewise distance *D_piecewise_* (*X_u_*, *Y_u_*) can be normalized by $D_{\text{piecewise}}^{\text{norm}}(X_{u},\;Y_{u})=D_{\text{piecewise}}(X_{u},\;Y_{u})/2$. Usually we may take *ρ* = 3.

## 4. Evaluation of the Proposed Distance Metrics from the Biological Point of View

We propose to replace the pseudo-distance in the dynamical language approach [5] by the chord distance or piecewise distance. We need to examine the effects of this replacement from the biological point of view. In order to do this, we evaluate the new distance metrics on four datasets, namely **Dataset 1** of 109 complete genomes of prokaryotes and eukaryotes used in [11], **Dataset 2** of 34 prokaryote and chloroplast genomes used in [12], **Dataset 3** of mitochondrial genomes of 64 selected vertebrates used in [19], and **Dataset 4** of 62 complete genomes of alpha-proteobacteria used in [31]. (*Note*: Chan *et al.* [21] recently tested the chord distance with different denoising formulas on Dataset 2).

We used the dynamical language approach for Datasets 1 and 2 in [5] and Dataset 3 in [32]. Some biological comparisons of this approach with the Markov model approach on Datasets 1 and 2 were given in [5]. Recently we found that wrong data of the Archaea Crenarchaeota bacterium *Pyrobaculum aerophilum* (Pyrae) from Dataset 1 was used in [5]. Using the right genome data, *Pyrobaculum aerophilum* (Pyrae) groups with the other Archaea Crenarchaeota bacteria correctly (when we use the amino acid sequences of all protein-coding genes from genomes and *K* = 6). After this correction, the resulting tree is better than the one in [11] from the biological point of view, with all firmicutes group together and the other branches are similar. For Dataset 2, we obtained two trees with the same topology to those using the dynamical language approach in [5] and the Markov model approach in [12] (also using the amino acid sequences of all protein-coding genes from genomes and *K* = 6). For Dataset 3, we reported in [32] a good tree in agreement with the current understanding of the phylogeny of vertebrates revealed by the traditional approaches using the dynamical language approach (based on the whole DNA sequences of genomes and *K* = 11). This tree is better than the one in [19] and the one obtained by the Markov model approach. Hence we just need to compare the best trees obtained by the dynamical language approach using the two proper distance metrics with the best trees obtained from the pseudo-distance in [5] based on the first three datasets. In 2009, Guyon *et al*. [31] compared four alignment free string distances for complete genome phylogeny using Dataset 4. We will compare our method in this paper with the results in [31] based on Dataset 4.

**Figure 2.**
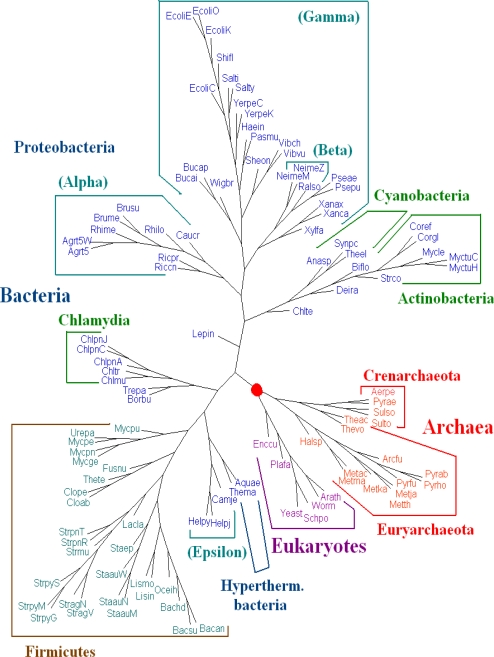
Phylogeny of 109 organisms (prokaryotes and eukaryotes) using the dynamical language approach with chord distance in the case *K* = 6 based on all protein sequences.

The whole DNA sequences (including protein-coding and non-coding regions), all protein-coding DNA sequences and the amino acid sequences of all protein-coding genes from genome data are used for phylogenetic analysis. For **Dataset 1**, we have seen that amino acid sequences of all protein-coding genes from genomes give better results than those given by the whole DNA sequences and all protein-coding DNA sequences. We evaluated the dynamical language approach with chord distance and piecewise distance on the amino acid sequences of all protein-coding genes from genomes for *K* = 3, 4, 5 and 6. We find the trees using the new distance metrics have the same topology as the trees using the old “distance” for the same value of *K*, and the trees for *K* = 6 are the best. Here we present the tree for *K* = 6 using dynamical language approach with chord distance in Figure 2. The phylogeny shown in Figure 2 supports the broad division into three domains and agrees with the tree of life based on 16S rRNA in a majority of basic branches. For further biological discussions, one can refer to [5] with the correction for the position of *Pyrobaculum aerophilum* (Pyrae).

**Figure 3.**
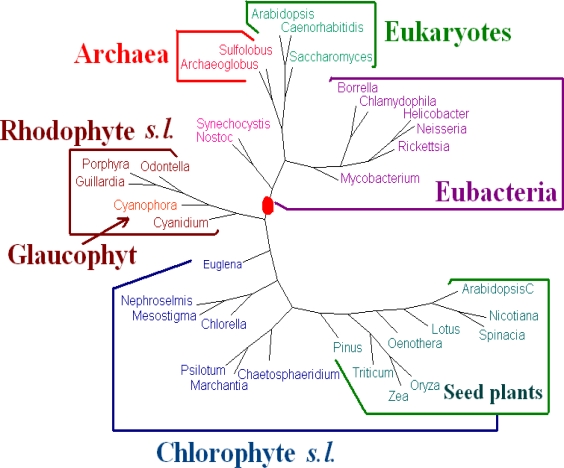
Phylogeny of chloroplast genomes using the dynamical language approach with piecewise distance in the case *K* = 6 based on all protein sequences.

For **Dataset 2**, we have seen that the amino acid sequences of all protein-coding genes from genomes give better results than those given by the whole DNA sequences and all protein-coding DNA sequences. We evaluated the dynamical language approach with chord distance and piecewise distance on the amino acid sequences of all protein-coding genes from genomes for *K* = 3, 4, 5 and 6. We find the tree using the piecewise distance has the same topology as the tree using the old “distance” for the same value of *K*, the tree using the chord distance has similar topology (a little bit worse because *Pinus thunbergii* is separated from its correct position) to the tree using the old “distance” for the same value of *K*. And the trees of *K* = 6 are the best. Hence we present the tree for *K* = 6 using the dynamical language approach with piecewise distance (*ρ* = 3) in Figure 3. We also note that the topology of the tree in Figure 3 is the same as that of the tree obtained by the Markov model in [12]). The phylogeny of Figure 3 shows that the chloroplast genomes are separated to two major clades corresponding to chlorophytes *s.l.* and rhodophytes *s.l.* The interrelationships among the chloroplasts are largely in agreement with the current understanding on chloroplast evolution. For further biological discussions, one can refer to [12].

**Figure 4.**
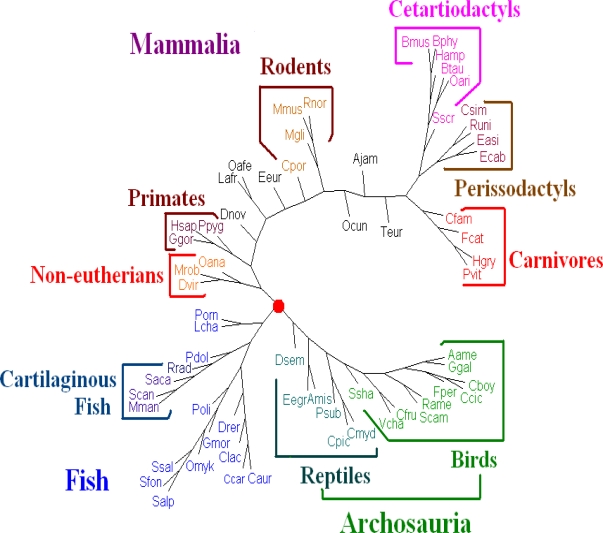
The NJ tree of mitochondrial genomes based on the whole DNA sequences using the dynamical language approach with chord distance in the case *K* = 11. In this tree the birds and reptiles group together as Archosauria.

For **Dataset 3**, after comparing all the trees with the traditional classification of the 64 vertebrates (the traditional classification from the KEGG database is available under “Complete Mitochondrial Genomes” on http://www.genome.jp/kegg/genes.html)), we find that the whole DNA sequences give better results than those given by the amino acid sequences of all protein-coding genes from genomes and all protein-coding DNA sequences. We evaluated the dynamical language approach with the proposed distance metrics on the sequences of whole genomes for *K* = 6 to 13. We find the tree using the piecewise distance has the same topology as the tree using the old “distance” for the same value of *K*, the tree using the chord distance has similar topology (a little bit better because *Dasypus novemcinctus.*(Dnov) is close to but does not remain in a branch of primates) to the tree using the old “distance” for the same value of *K*. And the trees for *K* = 11 are the best. Hence we present the tree for K = 11 using the dynamical language approach with chord distance in Figure 4. The tree (Figure 4) generated is similar in topology to the tree obtained using the SVD method in the case *K* = 4 [19], and is also similar to a recently generated tree of 69 species [33], placing a vast majority of species into well-accepted groupings. As shown in Figure 4, our distance-based analysis shows that the mitochondrial genomes are separated into three major clusters. One group corresponds to mammals; one group corresponds to the fish; and the third one represents Archosauria (including birds and reptiles). The interrelationships among the mitochondrial genomes are roughly in agreement with the current understanding of the phylogeny of vertebrates revealed by the traditional approaches. For further biological discussion, one can refer to [32].

**Figure 5.**
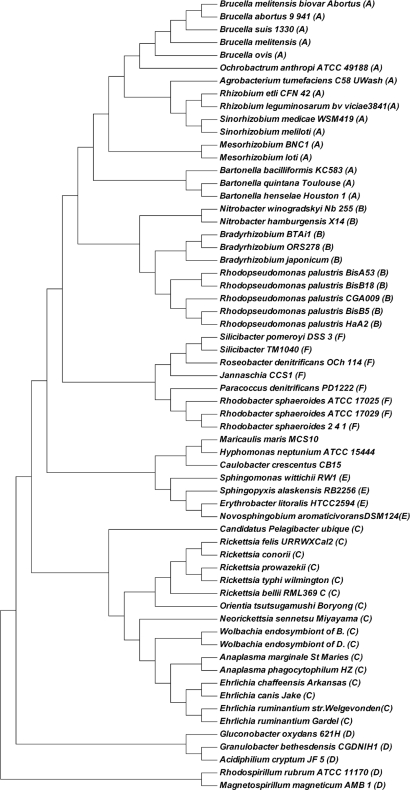
Phylogeny of 62 alpha-proteobacteria using the dynamical language approach with chord distance in the cases *K* = 5 and 6 based on all protein sequences. The topology of trees obtained by the dynamical language approach with pseudo-distance in [5] and piecewise distance in the cases *K* = 5 and 6 based on all protein sequences are the same as that in this figure.

For **Dataset 4**, Guyon *et al*. [31] first reconstructed a reference tree using Maximum Likelihood (ML) method based on the large (LSU) and the small (SSU) ribosomal subunits sequences (*i.e.*, the traditional alignment method). Then they compared the results using four alignment free string distances for complete genome phylogeny. The four distances are Maximum Significant Matches (MSM) distance, *k*-word (KW) distance (*i.e.*, the Markov model in [11]), Average Common Substring (ACS) distance and Compression (ZL) distance. Guyon *et al*. [31] found the MSM distance out performs the other three distances and the KW cannot give good phylogenetic topology for the 62 alpha-proteobacteria (see Figure 3 in [31]). We tested our dynamical language approach with pseudo-distance in [5] and the two proper distances in this paper on Dataset 4. We found that amino acid sequences of all protein-coding genes from genomes give better results than those given by the whole DNA sequences and all protein-coding DNA sequences. We evaluated the dynamical language approach with pseudo-distance in [5] and the two proper distances in this paper on the amino acid sequences of all protein-coding genes from genomes for *K* = 3, 4, 5 and 6. We found the trees using the new distance metrics have the same topology as the trees using the old “distance” for the same value of *K*, and the topology of trees for *K* = 5 and 6 are the same and the best. Here we present the tree for *K* = 6 using dynamical language approach with chord distance in Figure 5. As shown in Figure 5, all Rhizobiales (Bartonellaceae, Brucellaceae, Rhizobiaceae and Phyllobacteriaceae) (A), Rhizobiales (Bradyrhizobiaceae) (B), Rickettsiales (Rickettsiaceae and Anaplasmataceae) (C), Rhodospirillales (D), Sphingomonadales (E); Rhodobacterales (Rhodobacteraceae) (F) group into correct branches respectively. Even inside each lineage (groups A to F), our phylogentic topology is more similar to that of ML reference tree (the right side tree in Figure 1 of [31]) than that obtained by the MSM distance (the best result in [31]). After comparing our Figure 5 with the tree obtained using KW distance (*i.e.*, the Markov model in [11]) (the tree in Figure 3 of [31]), our dynamical language model performs much better than the KW distance.

There is no significant effect by the normalization of the distances and different values of *ρ* ≥ 3. Using the proposed distance metrics, we compared the trees before and after normalization and found that the topology of the trees is the same. Then we set *ρ* = 4, 6, 8, 10 and found that we could get the trees with the same topology as the tree for *ρ* = 3. As a result, there seems to be no noticeable effect by normalization of the distances and different values of *ρ* ≥ 3.

## 5. Conclusions

We proposed two new mathematically proper distance metrics based on the lengths of the chords constructed from unit vectors and on proportions of the sample correlation function of unit vectors to replace the pseudo-distance in the dynamical language approach [5]. The results showed improvements with this replacement from a biological perspective. These results confirm their usefulness in phylogenetic analysis.
